# Social prescribing training for doctors: perspectives from people with lived experiences

**DOI:** 10.24095/hpcdp.44.6.10

**Published:** 2024-06

**Authors:** Herb Paquette

**Affiliations:** 1 Canadian Institute for Social Prescribing at the Canadian Red Cross, Mississauga, Ontario, Canada

**Keywords:** social prescribing, physician training, medical school, advocacy, health care cost reduction, superior health care

**Highlights**

- To move social prescribing forward, doctors need to know the value of this practice and where to prescribe in an easy way.
- Upon a visit to my physician, I learned that family doctors sometimes lack knowledge of the various agencies and organizations that can provide social prescribing, which is why they are unable to find appropriate social prescriptions for patients.
- My explorations made me realize that medical schools in Ontario might not be teaching social prescribing as a form of patient care in the way we define it.
- If we are to move social prescribing forward in Canada, we need to start at the beginning by having medical schools and relevant ministries of postsecondary education incorporate social prescribing into the curriculum.
- If medical schools would act as torch bearers for social prescribing, perhaps, as they do in other countries, our doctors would learn the value of providing and connecting patients to social support, in terms of both superior health care to patients and decreasing costs.

Dear Editors,

As someone with firsthand experience of how much social prescribing can improve health, I think it is of the utmost importance to move social prescribing forward. To achieve that, doctors need to know the value of this practice, where to direct patients, and how to do it in the easiest, most efficient way. Along the same lines, I believe that education in universities and medical schools is critical, because if students are not aware of social prescribing, they will not practise it or advocate for it.

I was recently visiting my family doctor, as I frequently do. I asked her if the term was familiar. She acknowledged it was, but not part of her practice, because she lacked knowledge of the various agencies and organizations that she might find appropriate to prescribe for her patients. I then asked my hematology doctor if he had ever heard the term. He replied “No,” and volunteered to do some research into it, but made “no promises.”

All this made me wonder if social prescribing was something taught in our medical schools. I did some rudimentary exploration on social prescribing training in medical schools. What my explorations seem to indicate is, it appears that there is not a lot of information on how much medical schools in Ontario might be teaching social prescribing as a form of patient care in the way we define it. As a disabled person, I have firsthand experience with being involved in a not-for-profit and the recipient of social prescribing dedicated to my health issue and my well-being, and there is no question in my mind they kept patients out of hospital beds with their (much) lower-cost service.

If we are to move social prescribing forward in Canada, we need to start at the beginning by having medical schools and relevant ministries of education incorporate social prescribing into the curriculum. The leading medical schools are highly influential, and if they lead by example by incorporating social prescribing into the curriculum, others will follow suit. 

If the medical schools would serve as torch bearers for social prescribing, as they do in other countries, perhaps our doctors would learn the value of providing and connecting patients to social support, in terms of both superior health care to patients and decreasing costs. 

I wholeheartedly believe that social prescribing is an important aspect of our health care that deserves more attention in family doctors’ and specialists’ offices, in medical school curriculums, in hospitals and in community, so that we can all work together towards building a healthy nation. 

Thank you.

Herb Paquette

